# Social and Psychological Capital for the Start-Up of Social Enterprises With a Migratory Background

**DOI:** 10.3389/fpsyg.2020.01177

**Published:** 2020-06-23

**Authors:** Camilla Modesti, Alessandra Talamo, Giampaolo Nicolais, Annamaria Recupero

**Affiliations:** ^1^Department of Social and Developmental Psychology, Faculty of Medicine and Psychology, Sapienza University of Rome, Rome, Italy; ^2^Department of Dynamic and Clinical Psychology, Faculty of Medicine and Psychology, Sapienza University of Rome, Rome, Italy; ^3^Department of Social, Political and Cognitive Sciences, University of Siena, Siena, Italy

**Keywords:** psychological capital, social capital, entrepreneurship, social integration, refugee

## Abstract

The highest number of forcibly displaced people has been currently recorded due to war, poverty, and climate change. Recently, a process that recognizes refugees as reliable interlocutors for the improvement of reception policies has started. Refugees are therefore encouraged to start up social enterprises aimed at fostering newcomers’ social integration to participate to such a phenomenon. Positive Psychology, with its focus on human strengths, allows to identify the resources that pushed refugees to turn the difficulties they faced during the journey and the resettlement process into resources for themselves and for the resettlement community. The following paper explores in particular the interplay between social and psychological capital that is at the base of a similar social entrepreneurship project through a case study. A qualitative research has been carried out within a social enterprise with a migratory background to analyze the internal and relational resources that brought founders to start up the venture. Results show that while social and psychological capital were independently activated to start from scratch in the resettlement community, they occurred in interrelation in a subsequent phase when participants transformed their direct experiences related to migration into the human capital of their enterprise.

## Introduction

According to the United Nations High Commissioner for Refugees (UNHCR), there are 70.8 million forcibly displaced people worldwide: 41.3 million of them are internally displaced, 25.9 million are refugees, and 3.5 million are asylum-seekers. In 2016, in Europe, there were 2,268,993 refugees and 1,148,856 asylum seekers.

Even though such a population is commonly considered as beneficiary of public initiatives that address their social integration in the reception countries, nowadays, a process of recognition of refugees as reliable interlocutors for the improvement of the reception policies has started. Evidence of such a process are the constitution of the Global Refugee Forum within the Global Compact on Refugees lead by UNHCR^1^ and the release of calls addressed to refugee-led associations with the aim to foster newcomers’ social integration. These initiatives push refugees to start up social enterprises that, differently from nonrefugee-led organizations, take advantage of a direct experience about the effectiveness of the current social integration systems.

Refugees are therefore called to turn their first-hand experiences related to their social integration path into professional competences with the aim to foster newcomers’ social integration. The experiential knowledge developed through their own inclusion process in the resettlement community is therefore transformed into a personal resource, as it drives a professional development, as well as into a collective resource. When integrated within the resettlement community, migrants participate indeed to its social, cultural, and economic development.

The research is therefore based on a problem framing that considers todays’ migration issues not in terms of barriers or negative effects, but rather highlighting their function in fostering the cultural and social development of the reception countries. Studying the internal and relational resources that brought people with migratory background at starting up social enterprises for newcomers’ social integration allows to better address international top–down strategies, community interventions, and bottom–up ventures that address such a relevant issue.

## Theoretical Framework

By means of a Positive Psychology framework, this paper explores which personal and social resources allowed entrepreneurs with a migratory background to turn their personal experiences related to the integration within the resettlement community into a social entrepreneurship. A case study on the start-up of a social enterprise with migratory background (SEMB) will be presented.

### Positive Psychology and Social Integration

In 2000, Martin Seligman and Csikszentmihalyi (2000) published “Positive Psychology an Introduction,” which proposed a new theoretical approach in psychology interested at exploring the development of human inner strengths. According to the authors, after the Second World War, psychology had developed strictly around pathology, improving knowledge and competences in treating mental illness. At the same time, it also produced a victimization of human beings who became represented as passive spectators of external events. With the aim to “catalyse a change in psychology from a preoccupation only with repairing the worst things in life to also building the best qualities in life” (Seligman, 2002, p. 3), Seligman proposed a new perspective that investigates human strengths and virtues at a subjective, individual, and community level. Such a branch of studies is in continuous evolution producing not only theoretical but also practical advances. Positive Psychology has indeed guided several researches on the design of technologies to improve life conditions of vulnerable population (Talamo et al., 2011, 2017; Giorgi et al., 2013; Recupero et al., 2018).

As Cobb et al. (2019) have highlighted, the majority of psychological studies within the migrant field adopt a pathological model that investigates the negative effects of the adversities migrants face before, during, and after their resettlement. Life threats faced in the country of origin and during the journey represent significant stressors. This comes in addition to unstable life conditions in the hosting country that comprehend also job insecurity once the resettlement process has been fulfilled (Chirumbolo et al., 2017). Nevertheless, there is relevant literature focused on the inner resources that forcibly displaced people develop after the experience of adverse events and life conditions, as the constructs of resilience (Southwick et al., 2014), posttraumatic growth (Tedeschi and Calhoun, 2004), and the adversity-activated development (Papadopoulos, 2007) testify.

Along the same line, Cobb et al. (2019) sustain the need for a Positive Psychology of Immigrants that goes beyond the experience of traumatic events and should instead be focused on migrants’ internal and external resources that sustain their sociocultural adaption in the resettlement community. According to the authors, factors such as “national conditions of pre- and postmigration societies, acculturation processes, community contexts, family contexts, cultural values, and character strengths” (Cobb et al., 2019, p. 621) allow migrants to face the challenges brought by the migration and integration process. As the authors suggest, the resources that ease migrants’ social integration are therefore in the interaction among contextual, relational, and their personal characteristics.

Within this framework, the foundation of SEMBs raises a particular interest. Mair and Noboa (2003) underlined that the exposition to social issue is the trigger condition that brings people to start up social enterprises. In the case of SEMBs that deal with newcomers’ social integration, founders make a capital out of the internal and relational resources that have fostered their own social integration, in order to ease the same path that people arrived after them will inevitably cross.

In this paper, we particularly address the social and psychological resources that allowed founders of SEMBs to get integrated within the reception country and to start up their organization.

### Social and Psychological Capital

Literature commonly addresses resourceful social relationships as social capital. Although it developed more than a century before Positive Psychology, social capital is kindred to Seligman’s approach as it investigates resources that human beings produce when engaged in valuable relationships.

Pierre Bourdieu (1985), who is considered the father of this construct, defined such form of capital as “the aggregate of the actual or potential resources which are linked to possession of a durable network of more or less institutionalized relationships of mutual acquaintance and recognition—or in other words, to membership in a group—which provides each of its members with the backing of the collectivity-owned capital, a “credential” which entitles them to credit, in the various senses of the word” (p. 51). As the definition specifies, social capital is developed through the participation to a network.

Differently from Bourdieu who considered resourceful just the in-group connections, Putnam (1993) sustained that also the ties between people who belong to different social groups can produce resources. For this reason, the author proposed a differentiation between bonding social capital that is referred “to trusting and co-operative relations between members of a network who see themselves as being similar in terms of their shared social identity” and bridging social capital that “refers to relations of respect and mutuality between people who know that they are not alike in some socio-demographic (or social identity) sense (differing by age, ethnic group, class, etc.)” (Szreter and Woolcock, 2004, pp. 654–655). Linking social capital has been further added to address respectful relationships that connect people with asymmetrical power positions (Woolcock, 2001).

Nevertheless, as Portes (1998) highlighted, the mere embeddedness in a social network is not sufficient in order to exploit the capital it potentially creates. Internal resources are also required in order turn social resources from potential into effective. In this regard, the construct of psychological capital is useful to better understand how such a switch is made.

Within the Positive Organization Behavioral Framework, psychological capital has been proposed in 2004 by Luthans and Youssef in order to identify and improve those “human resource strengths and psychological capacities that can be measured, developed and managed for performance improvement” (p. 15). The authors emphasized that differently from personality traits, specifically addressed by the mainstream of the human resources management approaches, those capacities are state-like. Therefore, they can be developed through organizational interventions in order to improve managers’ and employees’ performance.

Moreover, the constituents of psychological capital are measurable so that its variation can be traced in order to inform about the effectiveness of the workplace interventions implemented. In the attempt to identify psychological capacities that could be at the same time state-like and measurable, Luthans and Youssef (2004) identified four basic elements of psychological capital:

– Confidence, developed from Bandura’s construct of self-efficacy, is defined as the persuasion on ones’ own ability to take advantage of personal skills to achieve goals in a given context (Stajkovic and Luthans, 1998 in Luthans et al., 2004);
– Hope is described by Snyder et al. (1991) as a positive motivational state resulting from an awareness about one’s own effective agency and ability to plan pathways to achieve goals (Snyder et al., 1991 in Luthans et al., 2004);
– Optimism is defined from Seligman’s conceptualization as “a positive explanatory style that attributes positive events to internal, permanent, and pervasive causes, and negative events to external, temporary, and situation-specific ones” (Luthans and Youssef, 2004, p. 17);
– Resilience is considered as the capacity to bounce back from negative events, uncertainty, or even overwhelming positive challenges.

The construct of psychological capital is close to the one of self-capital developed by Di Fabio (2014) and defined as “The positive self-evaluation of the self-concept characterized by one’s own ability to be committed, to identify significant objectives, to feel in control over life-events, to creatively solve problems, to change constraints into resources, to develop one’s own skills to apply decision-making skills to every aspect of life and to make decision carefully, vigilantly and adaptively” (Di Fabio and Duradoni, 2019, p. 2). While psychological capital is a repertoire of resources available in the present time and projected into the future, self-capital values the continuity of the individual’s experience from the past to the present.

Taken together, psychological and self-capital might explain how migrants turn their personal experiences in social integration and participation to social relationships into the human and social capital required to start up their SEMB.

### Starting Up a Social Enterprise With Migratory Background: The Role of Social and Psychological Capital

We define a SEMB as an enterprise started by migrants with different migratory backgrounds (asylum seekers, holders of work and study permit, second generation migrants) aimed at participating to the social and cultural development of the resettlement community. According to Mair and Noboa (2003), a social enterprise “is a set of interlocking opportunity-based activities by competent and purposeful individuals who—through their actions—can make a difference in society and are bounded by context” (p. 3). In this specific case, the SEMBs taken into consideration are the ones that pursue the social integration of newcomers.

As Berry (2003) proposed, the inclusion of migrants in a hosting community produces “acculturation” or rather an intercultural contact that is at the base of psychological and cultural changes both in migrants and in the resettlement community. According to Berry, acculturation is made of two processes: “cultural maintenance (to what extent are cultural identity and characteristics considered to be important, and their maintenance strived for); and contact and participation (to what extent should they become involved in other cultural groups, or remain primarily among themselves)” (p. 9). The author has defined four acculturation strategies that can be adopted by minority groups in dealing with the contact between their culture of origin and the one of the resettlement country (Berry, 1997): assimilation, separation, integration, and marginalization.

– Assimilation describes the behavior of people who do not wish to retain their heritage culture and seek daily contact with members of the dominant group.
– Separation defines instead the opposite process whereby people engage in interaction with people belonging to the same culture of origin and do not wish to keep in contact with other cultures.
– Marginalization describes the process whereby people do not engage with both culture of origin and others.
– Finally, integration is described as the desire to engage both with members belonging to the culture of origin and with those belonging to other cultures.

Such a model clarifies that integration is just one of the strategies migrants can adopt to adapt to the resettlement community; what differentiates it from the other strategies is the fact that migrants who pursue the integration strategy become experts of both their culture of origin and the one of resettlement. Such an expertise makes them cultural brokers (Jezewski, 1990) with respect to newcomers. In addition, this process produces also a first-hand knowledge about how to navigate between two different cultures beside an awareness about the needs and strengths of the current social integration policies of which they were the first beneficiaries.

Such a wealth of experience represents the human capital of SEMBs aimed at fostering newcomers’ social integration. Nevertheless, since the start-up of an organization is a challenging process, beside their human capital, entrepreneurs must rely also on a sturdy network of trustful and reciprocal relationship and on a solid psychological capital.

With regard to the first, in line with Granovetter’s (1973) theory on weak ties, Casson and Della Giusta (2007) suggested that an eclectic social network provides entrepreneurs with a broader range of information and, moreover, with information that they could not acknowledge from their same community of belonging. Trust, which is a specific feature of bonding relationships, is instead fundamental in a moment of resource acquisition such as economic capital and employees (Casson and Della Giusta, 2007). A strong and reliable network of resourceful relationships is particularly necessary for the foundation of social enterprises. Just because these kinds of entrepreneurship are aimed at solving social issues, they need to be embedded in the institutional and civic fabric whereby they operate (Evers, 2001). Because SEMBs are rooted in their entrepreneurs’ direct experience in social integration, the social networks that have fostered their integration path represents a potential capital for the enterprise, too, both to start up the venture and to achieve its mission. As Evers (2001) underlies, social enterprises indeed reproduce, maintain, and extend their social capital. Within the migration field, bonding and bridging social capitals are differentiated on the basis of the ethnic identity (Beirens et al., 2007), defined by Phinney et al. (2001) as “an individual’s sense of self in terms of membership in a particular ethnic group” (p. 496). For this reason, while bonding relationships address connections within people who belong to the same ethnicity or community of origin, bridging networks are instead referred to the relationships among people who belong to different ethnicities or communities of origin. Eventually, linking networks outline migrants’ linkages with institutions.

These different kinds of social relationships have been explored according to their role in promoting migrants’ social integration. Studies have highlighted that not-exclusive bonding relationships are a source of emotional, social support, and employment opportunities that in turn foster migrants’ bridging networks with the hosting community (Beirens et al., 2007; Calhoun, 2010; Eriksson et al., 2019). Bridging networks ease instead migrants’ access to services and information and an improved sense of security. Lastly, according Eriksson et al. (2019), linking networks are particularly resourceful for refugees as they get included in the reception system as soon as they ask for asylum. For this very reason, this kind of relationships provide the specific target of migrant population with housing, sanitary, educational, and professional support.

Just a few studies have explored the social capital that allowed migrants’ entrepreneurs to set up a venture in the resettlement community. Luthans et al. (2007) reported studies that testified the function of bonding networks not only in providing social and financial support but also in fostering the development of essential competences related to the start-up and management of a new venture. Similarly to Casson and Della Giusta, the authors specified also that bridging networks not only contribute to the start-up of the venture allowing migrant entrepreneurs to access new information, but they also contribute to its success by easing the acquisition of new customers (Luthans et al., 2007).

Literature suggests also that the creation of social capital to set up a new venture is linked to the founders’ ethnic identity. In a study carried out within migrant entrepreneurs resettled in Canada, Robertson and Grant (2016) found out a strong correlation between the exploitation of bonding coethnic social capital and the strength of the heritage cultural identity for the starting up of new ventures. On the contrary, the correlation between the creation of bridging social capital and Canadian identity was not significant even though a significant correlation emerged between the participation to the Canadian society and the construction of bridging social capital.

With regard to migrant entrepreneurs’ psychological and self-capital, Luthans et al. (2007) affirmed that just because they have already experienced the uncertainty linked to the migratory journey and to the establishment in a new community, migrants likely rely on a robust psychological capital to face the uncertainty that the start of a new venture requires (Luthans et al., 2007). Researches have indeed demonstrated that the start-up of a new business is associated with high level of self-efficacy and optimism in entrepreneurs. In particular, self-efficacy supports the identification and evaluation of opportunities and resources available (Boyd and Vozikis, 1994; Barbosa et al., 2007; Sequeira et al., 2007; Tsai et al., 2014) and reduces the risk perception and the probability of failure (Krueger and Dickson, 1994; Goel and Karri, 2006). Studies attested that optimistic people more likely start up new ventures (Dushnitsky, 2010) even with a reduced capital or in the need to find external funding (De Meza and Clive, 1996). Specifically regarding the start-up of social enterprises, so far, just a few studies explore the internal resources that drive the constitution of such a particular kind of entrepreneurship activity. According to Mair and Noboa (2003), self-efficacy is the internal resource that, together with empathy and moral judgments, drives the social entrepreneurs’ commitment toward the resolution of social issues. Later studies have confirmed the centrality of self-efficacy (Konakll, 2015; Aydoğmuş, 2019) and specified that a risk-taking attitude and perseverance are among self-efficacy constituents that motivate the foundation of a social enterprise (Chipeta and Surujlal, 2017).

Because psychological capital developed within the organizational psychology, just a few studies have explored such a construct in the migration field. According to Luthans et al. (2007), the motivations behind the migratory journey and the success of it are themselves clue of a high level of psychological capital in all its components. In addition, Cobb et al. (2019) suggest that resilience is a character strength that migrants can make use of in order to successfully go through the migration and resettlement process.

## Case Study

The case study presented hereafter explores the relationship between social and psychological capital experienced by migrant founders of social enterprises aimed at fostering newcomers’ social integration.

According to Mair and Noboa (2003), self-efficacy and social support together enable the start-up of social enterprises. Nevertheless, the relation between psychological and social capital is poorly investigated in the literature.

In their review, Newman et al. (2014) proposed that psychological capital moderates the engagement in social networks that are a source of emotional support, advice, knowledge, and information access. Fredrickson (2001) highlighted that people with high psychological capital tend to tie relationships within each other, increasing the chance to create alliances. At the same time, reliable social networks have the function to enhance those emotional competences related to psychological capital (Fredrickson and Levenson, 1998). Recently, Wang et al. (2019) found out a bidirectional relation among psychological capital and what they address as relational capital. In a qualitative research they carried out about the “effect of human, relational, psychological capital on new venture performance,” the authors found out not only that some components of psychological capital fostered the establishment of resourceful networks but also that some horizontal and vertical relationships nurtured entrepreneurs’ psychological capital.

Further investigation of such dimensions is needed to advance the knowledge about strategies and practice for migrants’ integration based on psychological and social capital.

To this end, the research has been carried out involving a social enterprise with migratory background established in Turin, in the North of Italy. The enterprise is made of four founding members and six associated members with different migratory backgrounds: refugees, holders of subsidiary protection, and second-generation migrants (Table 1). An Italian man is also one of the founding members. The funding members met through previous collaborations in projects of active citizenship and promotion of civil rights.

**TABLE 1:** Participants.

Age	M = 33.3, SD = 5.6
Gender	5M 4F
Nationality	3 Somali 1 Afghan 1 Pakistani 1 Italian-Peruvian 1 Romanian 1 Kashmiri 1 Italian
Migratory background	4 Refugees 2 Second generation 2 Subsidiary protection 1 None
Years in Italy	M = 15, SD = 10.08
Holders of Italian citizenship	4 members: 1 refugee, 2 second generation migrants, 1 Italian born

The enterprise has been started up in 2018 and participated to a UNHCR call aimed at sustaining migrant enterprises with a social impact acting at a community level in order to foster social cohesion and the integration and empowerment of refugees. Its mission is to promote and spread a culture of peaceful coexistence, dialogue and intercultural exchange and respectful of diversity, giving centrality to the role played by young refugees and new Italian citizens. In order to fulfill its mission, the enterprise implements several actions centered to the goal of creating bridges between refugees and migrants in general and members of the resettlement community. For this reason, projects aimed at fostering newcomers’ knowledge about the Italian history and culture have been implemented as well as sportive events such as football tournaments and a festival aimed at spreading the European solidarity values. In addition, training sessions about the Italian and European reception policies are implemented.

Beneficiaries of the enterprise are therefore not just refugees or migrants themselves but also members of the resettlement community who are interested in creating a cohesive society.

### Research Objectives and Methodology

Since it is a qualitative research, it is guided by two open-ended research questions that lay the groundwork of the data collection and analysis. The research questions (RQs) are the following:

RQ1: How do founders’ social and psychological capital sustained the creation of the SEMB?
RQ2: Do psychological capital and social capital interact fostering the start-up of the social enterprise?

The research has been carried out through an ethnographic investigation to analyze a context-specific case and derive rich description about the resources that entrepreneurs and associated members of the venture exploited in order to start the social enterprise and achieve its mission.

### Data Collection

Data have been collected from January to May 2019 by a Ph.D. student in social psychology supervised by two associate professors at the Department of Social and Developmental Psychology and at the Department of Dynamic and Clinical Psychology of Sapienza University of Rome. The Ethic Committee of the Department of Social and Developmental Psychology has released an ethical approval statement.

The aim of ethnography is to “represent otherness in such a way that “we” who are outside a relevant situation, can imagine what it is like to be in it” (Shweder, 1997, p. 18). Because the ethnographic approach is made of a multiplicity of investigation tools, data have been collected in the form of field notes, pictures, and narrative interviews.

Narrative interviews were employed as they allow to capture interviewees’ point of view and the meaning systems they refer to in order to interpret specific events (Atkinson, 1998). For this reason, it has been set up an interview framework made of open-ended questions in order to allow participants to describe their experience about the predefined topics.

The thematic areas explored through the interviews were (a) the motivations that pushed migration, (b) the social integration process, (c) the constitution of the enterprise, and (d) its goals, activities, and beneficiaries.

Nine members of the SEMB agreed to participate to the research and were interviewed. A total of 10 interviews were collected since the president of the enterprise was interviewed twice: beside the standard interview that has been submitted to all the members, he participated to a more detailed interview about the enterprise characteristics.

Participants were involved on a voluntarily basis without any payment or reward. They signed the informed consent that describes the research objectives and procedures and the collection and elaboration of data procedures in compliance with ethics and privacy regulations.

Interviews were performed face to face; they lasted for ∼40 min, and they were audiotaped. The audio records were coded with alphanumeric codes (ex., GP001) to preserve interviewees’ anonymity and then transcribed in Italian. In a second phase, they were translated into English.

### Data Analysis

Data analysis was performed through a “theoretical” thematic analysis (Braun and Clarke, 2006). Applying a sematic approach, data were initially organized according to the semantic content and then interpreted according to existing literature on social and psychological capital. A first analysis of the interview was conducted wherein the definitions of social capital and psychological capital were applied to extrapolate from the interviews the excerpts that better address these two constructs.

Three analytical categories emerged from this very analysis: social capital, psychological capital, and the interplay between social and psychological capital.

Then, a second analysis was done to differentiate between the three forms of social capital (bonding, bridging, and linking) and the four constituents of psychological capital (confidence, resilience, hope, and optimism). This was applied in order to differentiate among the components that constitute the two forms of capital. The different subcategories of social and psychological capital constitute the first level of subthemes in the thematical map.

The interviews were independently analyzed by two members of the research team and compared in a joint session to agree upon a shared interpretation in order to ensure validity and reduce as much as possible researcher bias.

## Results

Data show that both social and psychological capital played a significant role in participants’ social integration as well as in the foundation of the enterprise.

Within the narrations of participants’ resettlement path, the two analytical categories emerged as independent from each other. However, they emerged interrelated within the narration of the SEMB foundation. Such an interrelation created the third analytical category labeled “the interplay between social and psychological capital.”

### Social Capital

The type of social relationships resourceful in the resettlement phase seems to be linked to the stay permit received: participants who were not placed in reception centers at their arrival, mainly holders of subsidiary protection, relied mostly on Bonding networks, while holders of the refugee status who were instead placed in reception centers benefited mostly from linking networks.

The following excerpt is from a Somali men holder of the subsidiary protection. He arrived in Italy in 2003 by the age of 20. At first, he tried to cross the Italy–France border in order to continue his journey to the North of Europe, but because of the Dublin Regulation that imposes migrants to ask asylum in the Country of arrival, he was stopped and forced to remain in Italy.

Interview2-GP002

105. GP001: “So, I realized I had to start a new life in Italy and I started in Forlì in Emilia Romagna because there were some friends of my relatives’ who live in Sweden and they asked them to help me. So, I went to Forlì and then they brought me to the Caritas of Forlì that offered a bed to sleep.”

Such a result is of particular interests if connected to the functioning of the reception system. Going further in the interview, GP002 argued that when he arrived, the Italian reception system was not as prepared as it was by the time of the interview and for this reason, differently from the ones who obtained the refugee status, he could not be hosted in a reception center. Thus, he felt he had to build on his own his integration path in Italy. Therefore, in order to start this process, he first relied on people who belonged to his same community of origin, as they were the only acquaintances he had in the resettlement Country.

With regard to housing, being placed in reception centers as soon as they arrived, refugee interviewees reported that housing was not an issue they had to deal with at least at the beginning. Thus, they used the amount of time available before leaving the centers to become self-sufficient.

In this perspective, job represented the keystone. The following excerpt is from the interview of a Somali refugee woman of 32 years who arrived in Italy in 2013 by the age of 25. The role of linking networks in fostering job opportunities is highlighted.

Interview4-GP004

65. GP004: I created such a relationship with the educators that were there ((in the reception center)) so we created such a reciprocal trust and then we I learnt the language they tried to help me with job.

In the above excerpt, the participant tells about the beginning of her professional occupation as a cultural mediator. The presence of a trustful relationship with the educators of the reception center emerged as the trigger point for her job.

Differently from refugee participants, second-generation interviewees didn’t have to deal with housing. Because they arrived in Italy in the early infancy with their parents or reunited with them in the same life period, they used their school path in order to lay the basis for their participation to the Italian community. Since they were included in a context with Italian peers as soon as they arrived, the two second-generation migrant participants were the only one who could tie bridging networks from the very beginning of their integration path.

With regard to the rest of the group of participants, our results point out that they established bridging relationships in a second phase of their integration path, specifically once they had set the personal goals to achieve in the resettlement community. Therefore, bridging networks emerged resourceful precisely because they were oriented by specific objectives. For this very reason, the construction of bridging social capital emerged to be linked to psychological capital.

### Psychological Capital

Psychological capital is the second analytical category. Our results support the evidence whereby psychological capital plays an important role in the migration and social integration process as well.

With the exception of the beginning of the migratory journey that is clearly marked with hope, along the journey and the social integration path, the four psychological resources emerged as interrelated from our data. In particular, resilience emerge as a meta resource closely associated with the others. Evidence are reported hereafter.

The following excerpt reports the role of hope in pushing the President of the organization, a 32 years old former refugee man from Somalia in leaving his home country.

Interview8-GP001

4. GP001: If there’s anything that I am sure about, it’ s that if I overcame the difficulties it’s because being young and having the right to have a better future, I understood that this was not possible in Somalia, it was not possible. So I start from the assumption that where there is war it is not possible to have a good life, to have perspectives, at least in that period. However, I left apart my personal growth because when I left Somalia I was nineteen so I had to sacrifice myself mostly for my brothers than for me because they represented (.) let say a responsibility that I undertook. So I did this even for them. The specific goal that was really clear in my mind was to have a job, not matter what job but to have one in order to give them the chance to study and I have to say that now I am really happy to have given them at least a chance. Yes, this makes me really happy more than my personal path.

Here, the interviewee argument that, at the base of his flight, there was the need to have a job in order to accomplish the duty he assumed to guarantee a better future to his younger brothers, a duty that he could not accomplish in Somalia because of the war. Such a duty he felt became therefore the goal that drove his journey to Europe, where he expected to have the better future he deserved because of his young age. Consistent with the definition of hope (Snyder et al., 1991) that includes a sense of agency and a planification process, the resettlement at first and the job at second represented the path the interviewee designed to achieve his own goals.

Nevertheless, such a path brings along with it the difficulties related to the journey and resettlement venture. Hereafter, the role of optimism in giving to such a harsh period a transitional connotation is presented.

Interview8-GP001

47. *GP001:* There is an adage that says “If you lacked anything it was because it was not destined to you, you cannot lack what you should have had, know that victory comes with patience” this is a prophet’s saying that I’ve always used.
48. I: ***Is it a Muslim saying?***
49. GP001: A prophet’s saying that explains that life comes with patience so even difficulties come with solutions I mean if you have any difficulty it is not meant to be forever, so it is that moment that you don’t have to lose faith.
50. I: ***faith***
51. GP001: *Yes, you have to have the patience to overcome that moment it is not easy but to me it has been a great teaching that has been useful during the journey but also here (in Italy)*.

In the above excerpt, the President of the enterprise reports a life philosophy he directly relies on during adverse periods. Such a philosophy that comes from Islam, participant’s religious faith, encourages believers at considering difficulties temporary and holders of solution. In such a way, optimism here emerges as an attitude that fosters resilience as it allowed participant not to surrender but to face adversities.

Results attest that resilience is combined with confidence, too. In the following excerpt, their interplay is showed.

Interview4-GP004

38. GP004: At the beginning it was not easy because, you know, when you don’t know the language you can’t communicate and you can’t say what you want to say and it is really hard and then no one helps you and you are alone. And then in that moment I fought and I told myself “You will do it, you will do it” and eventually I found a job.

In the above excerpt the role of the participant’s confidence in supporting her motivation to the process of language acquisition in moments of discouragement because of the difficulties she met is recognizable. In particular, her sense of agency is identifiable. As aforementioned, such an excerpt highlights also that, under the disadvantageous conditions that migrants frequently go through, especially at the beginning of their integration path, confidence is strongly related to resilience. Such a resource can be indeed traced in her attempt to rely on her self-efficacy to navigate the difficulties she encountered.

Moreover, data show that a complementary relation exists between resilience and confidence, too. Evidence are in the following excerpt.

Interview6-GP006

52. GP006: Before I wasn’t like this, my life changed a lot when I lived for three-four months outside in a park. Before life to me meant buying clothes and branded shoes, spending money for useless stuff. When I passed this extremely hard period of my life, I found out that that wasn’t the real meaning of life. The meaning of life is completely different because in that moment I had nothing to eat, I had no place to sleep safely I couldn’t even have a shower safely, I bathed in the fountains. In that moment the idea the goal of my life changed completely. To me the sake of society is reached by helping poor people poor people in the territory. (…) What I was thinking is that there is still lot of work to do I am still doing nothing because I’m not well set, well organized to help many others. I do just a very little just the thing that I can do but I am not doing much.

The participant, a Pakistani man who arrived in Italy in 2015 by the age of 22, describes the harsh conditions he went through while waiting for the approval of his asylum request. Nevertheless, as he explains, these experiences represented a turning point in his life. Not only he coped with them, but he also used them as a wealth of experience to elaborate a new view of life. A shift in terms of commitment to the community is also traceable as a result of such a process. The interviewee highlights the individualistic approach to the community he had before the events he tells about. The harsh experience he went through fostered instead a collectivistic commitment to the resettlement community guided by the goal of helping poor people. Confidence, here intended as an awareness about one’s own skills in accordance with reality limits, is therefore identifiable, too. The man explains indeed that he would like to do more for poor people, but he is still not able to do it because he, in first person first, has to first get well settled within the community.

While resilience allowed participants to bounce back from the adversities they faced during the settlement phase, confidence emerge as the resource that allowed founders to turn these adversities into the wealth of knowledge that constitute the enterprise human capital.

### The Interplay Between Social and Psychological for the Start-Up of the SEMB

The “interplay between social and psychological capital” emerged as a third analytical category. It is made of contents whereby social and psychological capital are interrelated. Interestingly, their combination emerges specifically in relation to the start-up of the enterprise.

As aforementioned, resilience and confidence emerged as the essential ingredients for the creation of human capital out of participants’ direct experiences in resettlement. Through resilience, our participants managed to successfully navigate the adverse conditions they went through during the migratory and social integration process. Confidence was instead necessary to turn these experiences into skills and knowledge that could drive their personal and professional goals.

Evidence of this process are provided in the following excerpt whereby the establishment of resourceful relationships emerges as driven by psychological capital.

Interview1-GP001

18. GP001: People who arrive after us are going to meet our same difficulties, but some of them can already be overcome. It is not possible that the same difficulties I met ten years ago must be met by another person. Things that can also be trivial, very simple to solve. […] I said “I have to give some answers” but not on my own, rather creating alliances among Italians, guys from new and old generations. Together we can carry out concrete actions capable of creating bridges among different cultures.

In this excerpt, resilience is identifiable in the mentioned challenging situations that the interviewee dealt with during his resettlement. Confidence is instead traceable in his awareness about having the answers to some of the needs that newcomers may encounter during the resettlement, answers that come from his direct experience.

This example is paradigmatic because of the association between social and psychological capital. As the president of the enterprise well explains, an eclectic bridging network is needed in order to carry out some of the solutions to ease newcomers’ social integration.

Direct experiences as addressees of reception policies are not the only components of the founders’ human capital. Before starting up the enterprise, three out of four founders worked as delivers of social integration services provided by nongovernment organizations (NGOs). Those experiences provided the soil to create reliable relationships and professional competences that are at the base of the creation of the SEMB.

Hereafter, the production of social capital as a results of previous working collaborations is described.

Interview1-GP001

120. GP001: GP008 particularly and also GP007 we know each other since a long time, since two thousand and fourteen more or less, yes two thousand and twelve with GP008 through the campaign “I am Italy too”. GP008 was an activist but we got acquainted later. She gave me the first instruments on activism too because she worked in an Italian NGO (…) and organized exchange projects and then we went also to Greece and she involved me in lots of initiatives in school projects even in the last years. I found out that we have lots of things in common, lots of points of view.

In this way, the President describes the establishment of a reliable bridging network with another founder. The opportunity to work together allowed them to discover their commonalities and a common approach to address newcomers’ social integration, as he explained later in the interview. Such a shared vision represents an important ingredient for the start-up of the enterprise. Moreover, the relationship with the other founder provided him with professional skills that—together with a progressively acquired acknowledgment of the contextual framework whereby social integration facilities are run—complement the human capital coming from founders’ direct experiences in social integration.

The decisional process that brought founders to exploit such a wealth is described in the following excerpt whereby social capital emerges this time as the source of a collective psychological capital.

Interview7-GP007

61. GP007: The enterprise was born from such a strong and trustful relationship and somehow from the willingness, I mean, we really felt the fatigue of being someone else’s armed wing, I mean ((we were)) the good guys who were carrying forward the others’ enterprise while we, in our minds, in our convictions, in our exchanges, we knew we had some potential when we gave life ((to the enterprise)). I mean we knew we were already heard, we knew we had some chances, so we said “Why don’t we value everything we have done so far?” Even just because ((we were)) so active, so present. Personally, I have never back out even though I worked so many hours a day I have never been scared of making new commitments. This is a serious commitment to which I’m fully engaged because I know I am surrounded by people I trust with whom I am on the same wavelength I mean we have the same perspective, we are not scared about future and we are really investing to go toward future.

In the above excerpt that reports founders’ transition from the status of employee to the one of founders of a SEMB, the interplay between human, social, and psychological capital is clearly defined. Founders *valued* the common experiences collected during the course of time when three out four of them worked for someone else’s profit, to constitute the enterprise human capital. The valorization of the capital collected is explicitly mentioned by the interviewee. Nevertheless, according to the man’s words such a venture was possible because of *strong and trustful relationships* that tie the founders and constitute the enterprise bridging social capital. In the last line, he emphasizes again such a concept by saying that, because of trustful relationships characterized by the presence of common objectives and perspectives, the group of founders could *invest to go toward the future*.

These last words suggest that such a strong and reliable relationship is the source of a group confidence that developed through the course of time during chances of comparison and discussions where they realized they had some potentialities.

The social capital founders created over the course of time was essential no just to start up the enterprise but also to achieve its own mission which is to emphasize migrants’ role in the creation of inclusive communities. For this very reason, beneficiaries’ empowerment is a specific goal of the enterprise.

In the following excerpt social capital emerges again as a mean to develop confidence, in particular beneficiaries’ confidence.

Interview8-GP001-interviewerC

156. GP001: *((a beneficiary)) is here ((in Italy)) since five years now. I met him in the reception center where I worked. He is an exploited person, exploited, there are no other words. They had to be in a porter’s lodge, in a kitchen they had to be warehouse workers, they slept there and after five years they didn’t know anybody. This is not the point if you lose that job because you don’t accept anymore to work under that conditions*.
157. I: ***You have no chances***
158. GP001: Where do you go?
160. I: ***Alternatives***
161. GP001: Where do you go? Who knows you? Who do you know? So what? You start crying? No you don’t, you go outside and I ‘ll keep you in touch with some other people that I think can be an opportunity.

In the above excerpt, the President describes the process whereby reliable relationships with the founders of the enterprise are used to develop beneficiaries’ confidence. In particular, social relationships are here presented as the means to get out from a condition of labor exploitation that puts the beneficiary into a powerless position in terms of job access. The reliable relationship with the President is a means to empower the beneficiaries precisely because he can include them in other resourceful relationships. In such a way, the beneficiaries not only gain access to the social capital provided by the enterprise, but they are also supposed to develop psychological capital.

## Discussion

Literature on social entrepreneurship highlights the continuity between founders’ direct experience of a social issue and the foundation of such a specific kind of enterprise (Mair and Noboa, 2003). The resources that founders personally developed for the improvement of their own condition are indeed capitalized to start up an organization aimed at solving the same issue but at a social level.

The case study presented exemplifies such a process. The social enterprise under consideration has been indeed founded in order to use the wealth of experience and resources collected by its founders during their own social integration path, first to set up the venture and second to ease the same path of its beneficiaries. Interestingly, the foundation of the enterprise itself emerge as a mature stage of its founders’ social integration path, as it represents a concrete commitment toward the social development of the resettlement country.

The thematic analysis has shed light over an interesting dynamic about the development and use of the social and psychological resources that fostered founders’ integration. With regard to the narration of the very first stage of their integration path whereby participants had to face the necessity to get settled in the new country, social and psychological capital emerged as independent within each other.

With regard to social capital, consistent with the literature (Beirens et al., 2007; Calhoun, 2010; Eriksson et al., 2019), our data suggest that linking and bonding networks were fundamental in order to lay the groundwork, to provide the base to set up a new life in the resettlement community. In particular, those who were recognized as holders of the refugee status could benefit mostly form the linking capital. Similar to the results of Eriksson et al. (2019), our findings attest indeed that trustful relationships with the educators of the reception center eased participants’ job achievement. Those who could not benefit of the refugee status relied instead to the bonding relationships in order to start their new life in Italy. Consistent with the literature (Cheung and Phillimore, 2014), these kinds of network emerged as particularly useful for housing.

According to the literature, bridging networks are the hardest to be established because easily characterized by mutual distrust (Raghallaigh, 2013; Eriksson et al., 2019). Nevertheless, our results suggest that such a kind of relationships were tied at a later stage of our participants’ social integration, specifically when they started to plan their professional development. Bridging networks were indeed mentioned in association to objectives, and therefore, they appeared related to a sense of long-term perspective and agency. Because related to a self-achievement dynamic, bridging social capital emerged in interrelation with psychological capital.

Consistent with the limited existing literature on migrants’ psychological capital (Luthans et al., 2007; Cobb et al., 2019), our results suggest that participants developed and used their psychological capital to go through the migratory journey and social integration process. While hope and self-efficacy lead to planification processes, resilience and optimism sustained participants under difficulties. In particular, as pointed out by psychological literature in the migration field (Southwick et al., 2014; Cobb et al., 2019), resilience turned out to be the most prominent resource. Indeed, such a component of psychological capital turned out to be fostered by hope and optimism, while it is in a circular relationship with confidence. Resilience and confidence combined emerged as the internal resources that allowed founders to turn adversities into the human capital of their social enterprise. Consistent with previous studies (Boyd and Vozikis, 1994; Barbosa et al., 2007; Sequeira et al., 2007; Dimov, 2010; Tsai et al., 2014), confidence allowed participants to recognize their direct experience in social integration as acquired skills and knowledge that could be applied to drive the social development of the resettlement community.

Such a process is the keystone of the SEMB and combined with social capital occurred specifically in founders’ narratives about the creation of the enterprise. Consistent with the findings of Wang et al. (2019), our results attest indeed a bidirectional relationship between social and psychological capital in such a phase.

Interestingly, despite that the literature addresses bonding networks as the source of capital that provide migrants’ enterprise with social resources (Luthans et al., 2007), our results attest instead that bridging networks are the real core of the enterprise. Such a result is justified by the origin of the enterprise that comes from previous professional collaborations among the founders. In other words, founders met and started their collaboration because of common goals and skills that allowed them to progressively build a reliable relationship characterized by mutual trust and commitment. In line with the findings of Newman et al. (2014), in our case study, bridging social capital is the means that allowed founders to give expression to the human capital they developed through the aforementioned interplay between resilience and confidence. Nevertheless, along the same perspective of Fredrickson and Levenson’s (1998), social capital emerged also as a source of psychological capital. In particular, the trustful hallmark of the founders’ relationship developed a sense of collective confidence that allowed them to set up the enterprise. As previous studies highlighted (Casson and Della Giusta, 2007), social relationships provide indeed a safe context that allows founders to undertake the risk of starting up a venture.

Eventually, the interrelated nature of the relationship between social and psychological capital emerges also within the narration of the SEMB mission. According to the founders, the reliable relationships they tie with their beneficiaries are indeed aimed at fostering their empowerment through the inclusion in broader resourceful networks. Therefore, the enterprise itself is conceived as an incubator of social and psychological capital.

## Conclusion

The paper presents a qualitative research about the role of social and psychological capital in the start-up of a social enterprises with migratory background. To this end, the research investigates the current situation of a SEMB in the light of the process that lead to its development, considering the peculiarities of the founders’ personal experience as migrants.

Of course, the investigation of barriers and psychological disorders related to migration is needed to prevent negative consequences and promote the well-being. But with this paper, our attempt is to build on positive aspects of the experience: strengths and capabilities of people who engage themselves in a SEMB; bonding, bridging, and also linking relations that facilitate and support the integration in the resettled community.

Results about social and psychological capital—that allowed participants to get settled within the Italian community before the start-up of the venture—attest that robust relational and internal resources are needed in order to create the capital that will be invested to set up the enterprise.

While the literature on social capital is already consistent, our research enhanced knowledge on psychological capital by deepening the internal resources that sustain migrants’ journey and social integration path. Moreover, it also explored its interplay with social capital to sustain not just the start-up of a migrant-led organization but also the achievement of its mission. Our data highlight the process whereby the interaction between social and psychological capital allowed migrant founders of a SEMB to transform the adversities they went through during the migratory journey and the resettlement process into resources not just for their personal development but also for the one of the reception community.

In conclusion, this research contributes to advance the knowledge about the social value of a SEMB and the way it develops through the exploitation of its founders’ psychological and social capital. Our results support the emerging concept of migrants as reliable interlocutors for the improvement of the reception policies, and key actors in the development of successful integration initiatives.

The research presented in this paper is not without limitations. First of all, the case study is context specific, meaning that it represents an example of SEMB with its own mission and peculiarities; further researches shall shed light over the transferability of the study results.

Moreover, the research takes place in the Italian context where national regulations and services inevitably determine the resources available as well as pose barriers and constraints. Comparison case studies with SEMBs placed in other countries might highlight how the context influences the development and exploitation of internal and relational resources for the creation of this specific kind of enterprise.

As a future development of this research, we plan to investigate the psychological and social capital that the SEMB promotes in its beneficiaries for their social integration. This next step is intended to verify the effectiveness of the SEMB activities and the transferability of the internal and relational resources from the group of founders to their beneficiaries.

## Data Availability Statement

The datasets generated for this study are available on request to the corresponding author.

## Ethics Statement

The studies involving human participants were reviewed and approved by Ethical Committee of the Faculty of Medicine and Psychology of Sapienza University of Rome. The patients/participants provided their written informed consent to participate in this study.

## Author Contributions

CM, AT, GN, and AR drafted and reviewed the manuscript. All the authors provided approval for publication of the content.

## Conflict of Interest

The authors declare that the research was conducted in the absence of any commercial or financial relationships that could be construed as a potential conflict of interest.

## Footnotes

1 https://www.unhcr.org/global-refugee-forum.html
